# The role of breastfeeding and formula feeding regarding depressive symptoms and an impaired mother child bonding

**DOI:** 10.1038/s41598-024-62168-y

**Published:** 2024-05-19

**Authors:** Clara Carvalho Hilje, Nicola H. Bauer, Daniela Reis, Claudia Kapp, Thomas Ostermann, Franziska Vöhler, Alfred Längler

**Affiliations:** 1 Department of Pediatrics, Gemeinschaftskrankenhaus Herdecke, Herdecke, Germany; 2 Professorship for Integrative Pediatrics, Institute for Integrative Medicine, Witten/Herdecke University, Witten, Germany; 3 Institute of Midwifery Science, Faculty of Medicine, University of Cologne, Cologne, Germany; 4 Department of Psychology and Psychotherapy, Faculty of Health, Witten/Herdecke University, Witten, Germany; 5 Department of Obstetrics, Gemeinschaftskrankenhaus Herdecke, Herdecke, Germany

**Keywords:** Psychology, Paediatrics

## Abstract

Associations between depressive symptoms and breastfeeding are well documented. However, evidence is lacking for subdivisions of feeding styles, namely exclusive breastfeeding, exclusive formula feeding and a mixed feeding style (breastfeeding and formula feeding). In addition, studies examining associations between mother-child-bonding and breastfeeding have yielded mixed results. The aim of this study is to provide a more profound understanding of the different feeding styles and their associations with maternal mental health and mother-child-bonding. Data from 307 women were collected longitudinally in person (prenatally) and by telephone (3 months postnatally) using validated self-report measures, and analyzed using correlational analyses, unpaired group comparisons and regression analyses. Our results from a multinomial regression analysis revealed that impaired mother-child-bonding was positively associated with mixed feeding style (*p* = .003) and depressive symptoms prenatal were positively associated with exclusive formula feeding (*p* = .013). Further studies could investigate whether information about the underlying reasons we found for mixed feeding, such as insufficient weight gain of the child or the feeling that the child is unsatiated, could help prevent impaired mother-child-bonding. Overall, the results of this study have promising new implications for research and practice, regarding at-risk populations and implications for preventive measures regarding postpartum depression and an impaired mother-child-bonding.

## Introduction

Pregnancy and the birth of a child is an extraordinary and overwhelming event for many women. It is accompanied by intense positive emotions^1,2^. However, the birth of a child is associated with several changes and can cause intense stress^2,3^. *Peri- and postpartum depression* is defined as an episode of major depression that is associated with childbirth^4^. With a prevalence ranging from 10 to 15%—or even higher in lower- and middle-income countries—postpartum depression (PPD) is a widespread illness^5–8^. Its effects on maternal and the infant’s health, is a well-recognized issue^9^. Several studies have examined developmental disturbances in children of (severely) depressed mothers^10–17^, such as behavioral problems, lower math scores, or a sevenfold risk of depression during adolescence^16^. PPD is furthermore associated with an *impaired mother-child-bonding*^14,18–24^, which poses an increased risk of impaired emotional, social and cognitive development in the child^25,26^. *Mother-child-bonding* is described as a general tendency in the first weeks and months after birth to engage in social interaction with each other^27^. It is conceived as the maternal bond towards the child and the child's attachment towards the mother^28^. Disruption of this process has a long history in research^29^ and has been described as a disorder *of mother-child*-*bonding*^19,30^. The mother’s emotional response to the child is considered an essential symptom of bonding disorder and may reach the level of hatred and rage^29^. *Mother-child-bonding* disorders include a lack of maternal emotion to the extent that it can cause distress, irritability, hostile and aggressive impulses, pathological ideas, and outright rejection of the child^19^. Numerous further studies provide evidence for an association between PPD and *mother–child bonding* disorders^14,18–20,24,31^.

A possibility to prevent adverse outcomes such as PPD and an impaired mother-child-bonding might be *breastfeeding*. Previous studies found supportive evidence that (exclusive) breastfeeding prevents PPD and a poor mother-child-bonding^32–41^ and vice versa, as PPD is associated with early cessation of breastfeeding^42–44^. Numerous other studies provide evidence of an association between weaning and maternal depression^45–50^. In addition, 60% to 90% of women indicate breastfeeding difficulties, which may be an additional source of stress^51,52^. This can lead to a vicious cycle, as breastfeeding difficulties are an additional stressor for the mother and may promote depression^53–55^.

Studies examining associations between mother-child-bonding and breastfeeding, as well as attachment—which focuses more on how the *infant* builds up a relationship with its primary caregiver^56^—and breastfeeding, have found mixed results^37,38,57–61^. Keller et al. (2016) found that the *Postpartum Bonding Questionnaire* (PBQ) factor “anxiety about care” negatively predicted breastfeeding duration beyond six months^62^. Besides the educational level of the mothers, this was found to be the most important influential factor on the duration of breastfeeding in a regression analysis. Systematic reviews of the associations between breastfeeding and mother-child-bonding found that maternal sensitivity and secure attachment were the aspects of mother-child-bonding most often associated with breastfeeding^63,64^. Regarding the associations between breastfeeding difficulties and mother-child-bonding, emotional availability was found to be negatively correlated with objective (e.g. cracked nipples) as well as subjective breastfeeding difficulties (e.g. perceived insufficiency of milk supply^65^). In another study, secure attachment also appears to play an important role in breastfeeding continuation when breastfeeding difficulties occur^66^. In addition, breastfeeding confidence was found to be positively associated with higher levels of maternal attachment^67^. A study by Smith and Ellwood (2011) reported a descending pattern from exclusive to mixed to no breastfeeding and receipt of emotional care from the mother. The authors suggest that the time invested in breastfeeding and the associated close contact between mother and child strengthens the bond between the two. More time may also improve breastfeeding itself^68^. However, to our knowledge, no study so far differentiated exclusive, mixed and no breastfeeding patterns with regard to mother-child-bonding in particular.

Further research on the association of breastfeeding on PPD and mother-child-bonding may contribute in a meaningful way to the prevention of subsequent disorders and the reduction of long-term consequences for mother and child. The aforementioned influence of mother-child-bonding on breastfeeding behavior led us to include bonding as an independent variable in the study. The study design explained below led us to examine the influence of depressive symptoms in pregnant women and the influence of depressive symptoms in postpartum mothers, also as independent variables, in relation to the different feeding practices. The aim of this study is to examine whether PPD and poor mother-child-bonding are associated with different feeding practices (exclusive, mixed and no breastfeeding) in order to gain a better understanding of the associations between feeding practices and adverse psychological outcomes, such as PPD. In addition, to better understand such associations, we investigated the reasons for non-breastfeeding or adding formula in our study. We hypothesized that mothers who breastfed (exclusively) at the time point of three months postpartum would show significantly less symptoms of PPD and would report a better bonding towards their child, than mothers who did not breastfeed at that time.

## Methods

### Study design and procedure

The present work was embedded in a larger research project “Baby-Friendly and Breastfeeding” (in the following *BaSti-study*) and is a longitudinal observational cohort design. We recruited women between December 2017 and November 2019 vis-a-a-vis prenatal (participants were at least in their 16th week of pregnancy) as well as postpartum (three months after birth) via phone after the participants gave their informed consent. Recruitment took place before and after midwife consultations and gynecological examinations, delivery room information evenings and, for only postpartum surveys, through the hospital staff at the neonatal wards. Four pilot surveys were conducted prior to actual implementation. The participating hospitals were two university teaching hospitals in Germany. Inclusion criteria were delivery in one of the participating hospitals, a minimum age of 18 years and sufficient German language skills. Stillbirths or early postnatal death led to an exclusion of the study. The final study sample comprised *N* = 307. Of the total sample, 90 women (29.3%) were surveyed during pregnancy. To ensure that the conditions of the surveys were as comparable as possible, the procedure was always as follows: participants received (1) an information sheet, (2) the consent form, (3) a questionnaire on sociodemographic data and the test battery. Prior to the introduction of the EPDS, the following standardized introduction was presented to the participants by the person conducting the survey: " I would like to move on to topics that may be uncomfortable for you. If you do not wish to answer a question, you are of course free to do so. It is important for me to emphasize at this point that negative thoughts and feelings about yourself and the child can be common during and after pregnancy and are, to a certain extent, quite normal. However, it is possible for these feelings to persist for several days or weeks, leading to what is known as PPD. It is important to us that this topic receives more attention and recognition in the future and is not made taboo, as this leaves those affected alone with it". We filled in the answers given in the postpartum telephone survey in the questionnaire and sent it to the participants upon request.

### Approval

All research was performed in accordance with relevant guidelines. The participants proved written informed consent to participate in the study. This study was conducted in accordance with the Declaration of Helsinki. The Ethics Committee of the University Witten/Herdecke approved all components of the project 86/2017.

### Instruments

#### Edinburgh Postnatal Depression Scale (EPDS)

To assess the occurrence of depressive symptoms we used the German version^69^ of the 10-item self-report scale EPDS^70^ as a screening tool. The EPDS was originally developed as a screening instrument for the postnatal period^71^, but because it has been validated in numerous studies as an applicable tool for both pre- and postpartum assessment^72^, we included it in both settings. Each item is scored on a 4-point scale ranging from 0 to 3—with a higher sum score indicating more depressive symptoms^20,69^. Responses are based on the mental state over the past seven days^69^. The data revealed a Cronbach’s α of 0.82 (prepartum) and 0.79 (postpartum) in our sample.

#### Postpartum Bonding Questionnaire (PBQ)

To investigate mother–infant bonding, we used the German version of the self-report PBQ^19,23^, which assesses a mother’s feelings or behaviors toward her infant^73^. In contrast to the original version, which consists of 25 items and proposes a four factor solution, the German version of the screening instrument consists of 16 Items and proposes a solution with only one general factor “impaired bonding”^23^. The Likert scaling of the PBQ ranges from 0 (always) to 5 (never), with a higher sum score indicating impaired bonding^20^. In our sample, the internal consistency was α = 0.80.

#### Questionnaire “Feeling Comfortable During Pregnancy” (original: Schwanger Wohlfühlen)

To assess possible covariates such as subjective mental and physical well-being, coping during pregnancy and partner relationship/social support, we administered the questionnaire “Feeling Comfortable During Pregnancy”^74^ during pregnancy. The questionnaire was originally developed in German and consists of 38 items. They reflect the dimensions “*affective well-being*”, “*physical well-being*” and “*coping during pregnancy*”. Responses are given on a Likert scale ranging from 1 (does not apply at all) to 7 (fully applicable). In our sample, the overall internal consistency reached Cronbach’s α = 0.82 and for the three underlying dimensions affective well-being α = 0.82, physical well-being α = 0.42 and coping during pregnancy α = 0.66.

### Statistical analysis

All analyses were performed using IBM SPSS Statistics 27.0 (IBM Corp 2020). Decisions regarding the use of parametric or non-parametric tests are, besides the scale levels of the variables, based on the examination of outliers and the following assumptions: additivity and linearity, homoscedasticity, independence of residual terms and multicollinearity^75^. We tested normality with regard to sample sizes ˂ 30^75,76^. To use either parametric or non-parametric tests was decided for each test individually.

Hypothesis testing was conducted in two steps. First, *unpaired group comparisons* and *correlational analyses* were performed. All significant interactions with more than two groups were analyzed with post hoc tests. Due to different sample sizes, we chose to use Hochberg’s GT2 as a post hoc test^75^. Second, *logistic regression analyses* containing the significant results out of step one were performed. Because the regression analyses were hierarchical^75^, we focused primarily on the Akaike information criterion (AIC) to decide on a model. The final model of the regression analyses was bootstrapped (*N* = 1000 samples, 95% confidence interval), to ensure its explanatory value.

#### Potential covariates

Given previous research on the EPDS postpartum, the PBQ and the success of breastfeeding^20,23,77–81^, we included the following covariates (see Table 1) to minimize potential confounding.

**Table 1:** Included potential covariates in the analysis.

Potential covariate
Education
Previous psychiatric diagnoses
Number of children/whether the woman was primiparous
Parttime work/planned parental leave
Depression prenatal (Edinburgh Postnatal Depression Scale (EPDS))
Low control during delivery (emergency caesarean)
Caesarean section in general
Partner relationship/social support ("Feeling Comfortable During Pregancy" (original: Schwanger Wohlfühlen))
Prenatal medical care
Length of stay at the hospital
Mental wellbeing (“Feeling Comfortable During Pregnancy” (original: Schwanger Wohlfühlen))
Physical wellbeing (“Feeling Comfortable During Pregnancy” (original: Schwanger Wohlfühlen))
Coping mechanisms (“Feeling Comfortable During Pregnancy” (original: Schwanger Wohlfühlen))

## Results

### Descriptive statistics

Women were aged between 19 and 57 years (*M* = 32.93, *SD* = 4.50) and 87.6% of the women were born in Germany. 10.1% of the women have previously had a psychological illness. 12.7% of the children had to be transferred to the pediatric clinic after birth. See Table 2 for additional demographic characteristics.

**Table 2:** Descriptive statistics of the study sample divided into the different feeding groups (*N* = 307).

Variables						
*Feeding group*	Breastfeed	Weaned	Mixed feeding			
	77% (*n* = 236)	14% (*n* = 43)	9% (*n* = 28)			
*Hospital stay (in days)*^a^	*Mean*	*SD*				
Breastfeed	3.98	2.481				
Weaned	3.93	1.404				
Mixed Feeding	4.24	2.067				
*Prenatal care*	Gynecologist	Midwife	Both			
Breastfeed	49.2% (116)	0.8% (2)	50.0% (118)			
Weaned	60.5% (26)	0.0% (0)	39.5% (17)			
Mixed Feeding	57.1% (16)	3.6% (1)	39.3% (11)			
*Education*^b^	University degree	High school diploma	Secondary school degree	No degree		
Breastfeed	58.4% (136)	33.0% (77)	8.2% (19)	0.4% (1)		
Weaned	31.0% (13)	42.8% (18)	26.2% (11)			
Mixed Feeding	64.3% (18)	28.6% (8)	7.1% (2)			
*Number previous children*^c^	0	1	2	3	4	5
Breastfeed	55.6% (129)	33.6% (78)	7.3% (17)	1.8% (4)	1.3% (3)	0.4% (1)
Weaned	59.5% (25)	33.3% (14)	4.8% (2)	2.4% (1)		
Mixed Feeding	75.0% (21)	17.8% (5)	3.6% (1)	3.6% (1)		
*Previous psychological illness*^d^	Yes	No				
Breastfeed	10.8% (25)	89.2% (207)				
Weaned	9.5% (4)	90.5% (38)				
Mixed Feeding	7.1% (2)	92.9% (26)				
*Marital status*^e^	Married	Unmarried/Partnered	Separated/divorced			
Breastfeed	72.1% (168)	27.1% (63)	0.8% (2)			
Weaned	71.5% (30)	28.5% (12)				
Mixed Feeding	71.4% (20)	28.6% (8)				
*Birth mode*	Spontaneous deliver	Vacuum extraction	Primary caesarean section	Secondary caesarean section		
Breastfeed	58.1% (137)	11.8% (28)	9.3% (22)	20.8% (49)		
Weaned	51.2% (22)	9.3% (4)	20.9% (9)	18.6% (8)		
Mixed Feeding	57.1% (16)	14.3% (4)	7.1% (2)	21.5% (6)		
*Planned parental leave (in month*)^f^	*Mean*	*SD*				
Breastfeed	17.53	8.045				
Weaned	18.88	9.840				
Mixed Feeding	13.08	7.407				
EPDS postpartum (divided into the different feeding groups)	*M* = 4.91 *SD* = 3.806	*M* = 6.19 *SD* = 4.305	*M* = 6.43 *SD* = 4.646			
PBQ (divided into the different feeding groups)	*M* = 6.95 *SD* = 4.999	*M* = 6.14 *SD* = 5.060	*M* = 9.75 *SD* = 5.726			

^a^Up to 3.3% ( n = 10) missing values; ^b^up to ~ 1.3% (n = 4) missing values; ^c^up to ~ 1.6% (*n* = 5) missing values; ^d^up to ~ 1.6% (*n* = 5) missing values; ^e^up to ~ 1.4% (*n* = 4) missing values; ^f^up to ~ 16.3% (*n* = 50) missing values, *EPDS* Edinburgh Postnatal Depression Scale, *PBQ* Postpartum Bonding Questionnaire.

### Feeding styles and its associations with PPD and mother–child-bonding

A *t*-test did not reveal significant differences between the breastfeeding (n = 263) and the non-breastfeeding group (n = 43) concerning the postpartum EPDS, *t*(304) = 1.695, *p* = 0.09. Due to an outlier we found, we additionally performed a Mann–Whitney *U*-Test, which confirmed our results, *U*(43,263) = 4647.00, *Z* = −1.881, *p* = 0.060. However, a tendency was evident here. No significant difference could be shown with respect to PBQ scores either, *t*(304) = −1.320, *p* = 0.188.

Examining exclusive breastfeeding vs. mixed feeding vs. non-breastfeeding, an ANOVA revealed significant differences in EPDS scores postpartum, *F*(2, 304) = 3.323, *p* = 0.037, *r* = 0.145, with a small effect size^82^, as well as in PBQ scores, *F*(2, 304) = 4.689, *p* = 0.010, *r* = 0.173. For EPDS scores, post hoc tests did not reveal significant differences between the groups. For PBQ scores, post hoc tests showed significant differences between the breastfeeding group and the mixed feeding group, *M*_*Diff*_ = −2.801, 95% CI [− 5.24, − 0.37], *p* = 0.018, with *d* = 0.551, indicating a medium effect size^82^, as well as between the non-breastfeeding group and the mixed feeding group, *M*_*Diff*_ = *−*3.610, 95% CI [− 6.57, − 0.65], *p* = 0.011, with *d* = 0.677, also indicating a medium effect size^82^.

Based on these findings, we conducted multinomial regression analyses. Table 3 shows all correlations of potential variables relevant to exclusive breastfeeding. Significant correlations were included in the regression analyses. In order to obtain a feasibly parsimonious model, we decided to use a hierarchical method^75^.

**Table 3:** Results over variables relevant to exclusive breastfeeding.

Potential variables	Exclusive breastfeeding			
*Chi-square test*	*χ*^*2*^	*df*	*N*	*V*
Birth mode	12.089	8	307	.147
Primiparous	3.907	2	302	.114
Prenatal care	4.767	4	307	.088
*Spearman correlation*	*r*_*s*_	*p*	*N*	95% CI
Education	.178**^a^	.002	303	[0.063, 0.288]
Planned parental leave	− .045	.472	257	[− 0.170, 0.081]
Days of hospital stay	.070	.229	297	[− 0.048, 0.186]
Number children	− .079	.169	302	[− 0.194, 0.081]
EPDS postpartum	.141*^b^	.013	307	[0.026, 0.252]
EPDS prepartum	.227*	.032	90	[0.014, 0.419]
Quantity breastfeeding difficulties	.042	.460	307	[− 0.073, 0.157]
Affective wellbeing pregnancy	− .277**	.009	87	[− 0.465, − 0.064]
Physical wellbeing pregnancy	.034	.757	87	[− 0.184, 0.248]
Coping	.108	.314	89	[− 0.109, 0.315]

^a^**p ˂ .01, ^b^*p ˂ .05; *EPDS* Edinburgh Postnatal Depression Scale.

Our final regression model (Table 4) included the PBQ score and EPDS score prenatal as significant variables relevant to the different breastfeeding states (AIC = 148.514). The Likelihood Ratio test was significant, χ^2^(4, N = 86) = 17.647, *p* ˂ 0.001, and Nagelkerke’s R^2^ = 0.221 (Cox and Snell’s R^2^ = 0.184) can be considered as acceptable^83^. Both, Pearson test (*p* = 0.554) and deviance (*p* = 0.954), indicated that the model was a good fit to the data^75^. Bootstrapped regression weights (Table 5) confirm our results.

**Table 4:** Multinomial logistic regression model^a^ with breastfeeding states as the outcome variable (*n* = 87).

	Unstandardized *B*	Wald	*df*	*SE*	*p*	OR	95% CI
*Mixed feeding*							
PBQ	.224	9.134	1	0.074	.003**^b^	1.251	[1.082, 1.447]
EPDS pre	.080	0.824	1	0.088	.364	1.084	[0.911, 1.289]
*Non-breastfeeding*							
PBQ	.029	0.282	1	0.055	.595	1.030	[0.924, 1.147]
EPDS pre	.149	6.137	1	0.060	.013**	1.161	[1.032, 1.306]
*Model criteria*							
Nagelkerke’s R^2^	.221						
Cox and Snell’s R^2^	.184						
	χ^2^		*df*		*p*		
LRT (4, *N* = 86)	17.647		4		˂.001***^c^		
Pearson test	162.870		166		.554		
Deviance	136.514		166		.954		

^a^Reference category = exclusive breastfeeding; *LRT* likelihood ratio test; ^b^**p ˂ .01, ^c^***p ˂ .001; *EPDS* Edinburgh Postnatal Depression Scale, *PBQ* Postpartum Bonding Questionnaire.

**Table 5:** Confidence intervals and standard errors based on 1000 bootstrap samples (*N* = 90).

	*SE*	*p*	95% CI
*Mixed feeding*			
Constant	1.369	˂.001***^a^	[− 6.910, − 1.564]
PBQ	0.076	.004**^b^	[0.048, 0.340]
EPDS pre	0.104	.512	[− 0.170, 0.247]
*Non-breastfeeding*			
Constant	0.664	˂.001***	[− 3.586, − 1.044]
PBQ	0.067	.712	[− 0.124, 0.141]
EPDS pre	0.066	.007**	[0.041, 0.307]

^a^***p ˂ .001, ^b^**p ˂ .01; *EPDS* Edinburgh Postnatal Depression Scale, *PBQ* Postpartum Bonding Questionnaire.

We furthermore investigated *partner relationship/social support* for breastfeeding and *reasons* for non-breastfeeding or adding formula as well as associations with *breastfeeding difficulties*. Regarding partner relationship/social support, no significant difference was found between the groups, χ^2^(2, *N* = 200) = 3.293, *p* = 0.193. Table 6 shows the reasons given by the groups. Mothers who added formula reported more often than mothers who did not breastfeed, that they were unsure whether the infant was satiated/were sure that they were not satiated, and that the infant did not gain enough weight. In terms of *any* breastfeeding difficulties, correlations with the EPDS postpartum, η = 0.033, *p* = 0.568, and the PBQ, η = 0.077, *p* = 0.181, were both not significant. Regarding the *quantity* of breastfeeding difficulties no significant correlation was found with the EPDS postpartum, *r*(307) = 0.058, *p* = 0.311, 95% CI [− 0.054, 0.169], nor with the PBQ, *r*(307) = 0.080, *p* = 0.160, 95% CI [− 0.032, 0.191], either.

**Table 6:** Reasons for non-breastfeeding or adding formula nutrition.

Given reasons	Group mixed feeding^a^	Group non-breastfeeding^b^
I did not want to breastfeed long	0	3
I did not have sufficient milk	5	19
I was unsure whether my child was satiated	12	11
I did have a mastitis	2	3
My doctor advised me to	0	1
My midwife advised me to	1	2
Other breastfeeding difficulties	7	8
Illness	0	4
Medication	0	2
Dependency on breastfeeding	0	2
Tired of breastfeeding	0	2
Bottle-feeding is comfortable/as good as breastfeeding	0	1
Wanted to smoke again	0	2
Wanted to drink alcohol again	0	1
Child did not want to be breastfed anymore	0	1
For health reasons	3	3
Child refused the breast	0	7
Child got formula in the hospital	2	0
Stress	0	2
Child did not suck well	4	4
Insufficient weight gain of the child	7	3
Time aspects	0	1

Presented are the absolute frequencies*;* ^a^*n* = 22*;* ^b^*n* = 42.

## Discussion

The aim of this study was to investigate associations between depressive symptoms, mother–child-bonding and different feeding practices (exclusive, mixed, and no breastfeeding). We found that higher EPDS scores prepartum were associated with non-breastfeeding rather than with exclusive breastfeeding three months after birth. The association with the postpartum EPDS score was less clear. Nevertheless, an association between depressive symptoms occurring around pregnancy and birth and non-breastfeeding at three months after delivery was, as hypothesized, definitely reflected in our results and supports previous studies which found similar relations^36,40,46–48^. Another study that focused on the factor of a "breastfeeding method" found that exclusive or non-exclusive breastfeeding did not significantly affect depression, anxiety, or mother–child-bonding in the early postpartum period nor vice versa^84^. However the authors attributed these results to the short observation period of one month after birth, among other factors.

Considerations that PPD could be triggered by breastfeeding difficulties^54,55^ as a common source of stress^51,52^, are not supported by our results. According to Roth et al. (2021) breastfeeding difficulties are associated with lower bonding while postpartum depression has a negative impact on bonding quality regardless of breastfeeding difficulties: “The effect of breastfeeding difficulties on bonding persists over and above the effect of postnatal depressive symptoms”^85^. The authors concluded that postpartum depression and breastfeeding difficulties provide unique pathways for understanding bonding. A better understanding of bonding is relevant because studies have confirmed its importance, especially the importance of maternal sensitivity, for child development^86^. Furthermore, we found that with a higher PBQ score, mothers were signficantly more likely to report adding formula at the time point of three months than to exclusively breastfeed. To our knowledge, no study so far differentiated exclusive, mixed and formula feeding patterns with regard to mother-child-bonding in particular. Higher oxytocin levels may be of explanatory value for differences between exclusive and mixed breastfeeding. The quality of mother–child interrelations and maternal oxytocin levels appear to be positively correlated^87–90^. Oxytocin has also been found to be lower in patients with major depression^91^ and with higher depression symptoms postpartum^47,92^.

However, following the argument of oxytocin levels and breastfeeding, it remains unclear why mothers who had not breastfed also reported a better bonding quality than mothers who practiced mixed feeding, which is different from our hypothesis. Mothers who added formula reported reasons such as insufficient weight gain and non-satiation of the infant for doing so significantly more often than mothers who did not breastfed. Clayton et al. (2013) already found that infant hunger was a predominant reason for mixed feeding, rather than bottle-feeding alone^93^. In addition, no mother who added formula cited self-referred reasons, such as convenience, less dependency, stress or simply “not feeling like it” for their decision, whereas mothers who did not breastfeed did report such reasons. These results indicate that the underlying impulses and prior experiences that lead to the decision whether to add formula or to wean differ. First, women who decide to wean seem to be more likely to make decisions in their own favor, as the postpartum period has negative effects on their quality of life^94^. Second, these women may have more self-confidence to face potential stigmas associated with the role of the mother and (discontinued) breastfeeding. Hence, they may be able to see their bond with their child as unaffected by less successful breastfeeding. The relationship between self-confidence, self-care and bonding has been well investigated^95–97^ and may be of important explanatory value for our findings. Third, thinking that the child is not gaining enough weight or might be hungry could lead to feelings of guilt and cause stress. Postpartum stress decreases maternal self-confidence and affects the relationship between mother and child^98^. The decision to wean might be a relief for some women and reduce one source of postpartum stress. Symon et al. (2013) furthermore found the antenatal breastfeeding intention to be an important factor regarding satisfaction^99^. Maternal satisfaction scores were highest, when breastfeeding goals were reached, regardless of whether the goal was to breastfeed or intentionally not to do so^99^. Subsequently, regarding the given reasons to add formula, a possible explanation for higher PBQ scores of mixed feeding mothers in our sample could be, that they did not reach their breastfeeding goal, which subsequently resulted in a poorer rating of the bonding to their infant. However, more research is needed to prove our findings and conclusions, as the breastfeeding patterns, as distributed by us, and their association to mother–child-bonding, are to our knowledge still unexplored.

Our findings can be highly beneficial in terms of designing and disassembling preventive programs. They support for example indications to provide universal screening of previous depressive symptoms during pregnancy. It should be noted that screening in itself does not mean that depression can be definitively identified in an individual. Screening aims to identify as accurately as possible subpopulations with a high prevalence. A full diagnostic evaluation can only be made based on gold-standard clinical criteria^100^. Furthermore, peripartum maternal mental health is complex, as it comprises a variety of possible mental health problems^1^.

Screening should also be conducted postpartum, comprehensively, and with a special focus on at-risk populations. Especially reasons and impacts concerning a mixed feeding-style should be addressed in future research, to enable drawing more profound conclusions. Breastfeeding mothers with infants that do not gain enough weight or are not being sufficiently satiated, should especially be supported postpartum, as they tend to characterize their bonding as poorer, respectively. Education over these problems and possible solutions for them should get more attention during hospitalization and antepartum. According to our findings, mother–child-bonding seems to be an important variable in the issue of different feeding practices (exclusive, mixed, and non-breastfeeding). Depressive symptoms prepartum also seem to be an important variable in this context. We have not investigated to what extent the two are related. Although breastfeeding has many benefits for both the mother and the infant, the relationship between maternal mental health and breastfeeding is complex. It should be taken into consideration that breastfeeding may not be the most effective or viable option for all mothers^101^.

### Limitations

Several points limiting our findings should be considered. Although we included several possible covariates in our analyses, confounding factors might remain. We also did not impute the missing values (this refers especially to the prepartum data). When recruiting participants for the study, we were aware that this was a sensitive issue and that we did not want to commit to the sample size initially. We did not perform a power analysis prior to recruitment. Besides that, we did not evaluate our data with respect to dropouts. Hence, we cannot completely rule out an attrition bias. Future studies should collect data concerning information over their dropouts, as previous studies already reported for example higher depression rates in groups with missing data^16^. However, inclusion in our study was not very restrictive and data collection took place in different in-hospital environments. This led to a large sample that is representative of the facilities in which they were collected. The reliability of the subscales physical well-being and coping during pregnancy of the Questionnaire "Feeling Comfortable During Pregnancy" was lower than expected and not significant in the correlations (Table 3) and thus excluded from the regression analyses, which needs to be addressed here. Even though all screening tools used in our study were established and validated, it should be noted that we exclusively used self-report measures. These can cause biases, for example through answering in a socially desirable manner^102–104^, which could be crucial for our findings regarding associations between poor bonding and high education^23,81^. Reck et al. (2006) reported similar findings and hypothesized that higher educated mothers would have a greater tendency to answer in a less socially desirable manner, rather than actually suffering from poorer bonding to their infants^23^. Mixed-measure designs could prevent such biases. Future studies should for example consider including structured diagnostic interviews, video observations of maternal behavior or biochemical analyses. Depressed women furthermore tend to rate themselves as worse parents than they are, due to a low self-esteem and negative thoughts^105,106^, which could have affected some of the associations we found.

## Conclusion

Our findings provide important implications for future directions. These concern information regarding populations that may be at higher risk of developing PPD or a poor mother-child-bonding, such as antenatal depressive symptoms, or, representing to our knowledge a new finding, a mixed feeding style. However, more research is needed to get a more profound understanding of associations between mother–child-bonding and a mixed feeding style. Especially information about underlying reasons we found for performing mixed feeding, such as insufficient weight gain of the child or the feeling that the child was unsatiated, could contribute in a meaningful way to prevent impaired mother–child-bonding. We suggest implementing more measures supporting aspects such as self-efficacy and postpartum well-being of the mother in preventive programs.

## Data Availability

Due to the strict European General Data Protection Regulation the dataset generated in this study can not be deposited in a public repository. A request for access to the pseudonymized data for researchers who meet the criteria for access to confidential data must be submitted to the author: Clara Carvalho Hilje, email: Clara.CarvalhoHilje@uni-wh.de.
